# The cellular effects of novel triazine nitrogen mustards in glioblastoma LBC3, LN-18 and LN-229 cell lines

**DOI:** 10.1007/s10637-018-0712-8

**Published:** 2019-01-15

**Authors:** Rafał Krętowski, Danuta Drozdowska, Beata Kolesińska, Zbigniew Kamiński, Justyna Frączyk, Marzanna Cechowska-Pasko

**Affiliations:** 1 Department of Pharmaceutical Biochemistry, Medical University of Bialystok, Bialystok, Poland; 2 Department of Organic Chemistry, Medical University of Bialystok, Bialystok, Poland; 3 Institute of Organic Chemistry, Technical University of Lodz, Lodz, Poland

**Keywords:** Apoptosis, Glioblastoma, Hybrid anticancer drugs, Nitrogen mustard, Alkylating agent

## Abstract

1,3,5-triazine is an important heterocyclic skeleton for mono, two or three 2-chloroethylamine groups. The study presented here provides novel information on cellular effects of 1,3,5-triazine with mono, two or three 2-chloroethylamine groups in glioblastoma LBC3, LN-18 and LN-229 cell lines. In our study, the most cytotoxic effect was observed in 1,3,5-triazine with three 2-chloroethylamine groups (**12f** compound). It has been demonstrated that **12f** induce time- and dose-dependent cytotoxicity in all investigated glioma cell lines. Apart from that in glioblastoma cells, treated with **12f** compound, we noticed strong induction of apoptosis. In conclusion, this research provides novel information concerning cellular effects of apoptosis in LBC3, LN-18 and LN-229 cell lines. Moreover, we suggest that **12f** compound may be a candidate for further evaluation as an effective chemotherapeutic agent for human glioblastoma cells.

## Introduction

Glioblastoma multiforme (GBM) is one of the most common and devastating malignant tumor of human brain, characterized by local invasion and microvascular proliferation [1–3]. The most eminent feature of malignant gliomas is high resistance to chemotherapy and radiotherapy (RT), with an average survival less than 12–15 months, prolonged up to 3 years for less than 5% of patients only [4–6]. Current treatment options are based on the combined use of ionizing radiation and the DNA alkylating agent, a second-generation imidazotetrazine lipophilic prodrug - temozolomide (TMZ) [7–10]. Moreover, DNA-crosslinking agents, such as lomustine (CCNU) and nimustine (ACNU), are used as a second-line drugs. Their mechanism of action is based on O6-chloroethylguanine (O6ClG) generation, by chloroethylation of guanine in the O6-position, what finally leads to inter-strand crosslinks between N1-guanine-N3-cytosine (ICLs) [11–13].

There are known that difficulties in improving effective therapy in case glioblastoma are caused by very heterogeneous and anaplastic character of this cancer and are largely attributed to rapid growth and a high rate of recurrence [14, 15]. On the other hand, the response to anticancer drugs is frequently inhibited by a resistance to alkylating agents. O6-methylguanine-DNA methyltransferase (MGMT), which is a DNA repair protein, also remains a barrier preventing the successful treatment of patients with malignant glioma [16]. In turn, the functional role of family S100P proteins, which is associated with drug resistance and metastasis in many malignancies, has not been fully documented in glioblastoma [17]. Moreover it has been described that bis(2-ethylhexyl)phthalate (DEHP) modulates cell migration, invasion and anchorage independent growth through targeting S100P in LN-229 glioblastoma cells [18]. A recent work has suggested that podocalyxin (PODX), which is a highly glycosylated and sialylated transmembrane protein, promotes astrocytoma cell invasion and survival, despite of apoptotic stress, and contributes to GBM progression. Although there is evidence that PODX participates in epithelial-mesenchymal transition and interacts with different mediators of metastasis, the role of PODX remains to be elucidated [19–21]. *Liu* et al. reported that enhanced GBM cell invasion and proliferation is associated with fact that podocalyxin inhibits Ang-(1–7)/Mas signalling by down regulating the expression of Mas through a PI3K-dependent mechanism in GBM cells [22].

In view of the presented facts it is essential to develop new modalities of therapeutic approaches and identify therapeutic targets for advancements in malignant gliomas treatment.

The bifunctional alkylating agents, which include nitrogen mustards (NMs) e.g., mechlorethamine, chlorambucil, cyclophosphamide and melphalan, are used extensively since more than three decades in the treatment of autoimmune and neoplastic diseases [23, 24]. The nitrogen mustards present activity connected with their ability to inhibit the cancer cells proliferation by cross-linking the double strands of DNA. In addition, these compounds can inhibit DNA replication and transcription or lead to apoptosis and finally to the inhibition of tumor expansion [25].

It was demonstrated that 1,3,5-triazines substituted with mono-, di, and tri-[4-(2-chloroethyl)piperazin-1-yl] groups are strong alkylating agents, easily reacting with the most of nucleophilic functional groups, which are typical for proteins and nucleic acids [26]. It has been also reported that intensive structural modifications at 2-, 4- and 6- positions have allowed to obtain a wide group of structurally diversified derivatives associated with e.g. anti-cancer, anti-inflammantory, anti-bacterial properties [27–29]. Among various activities of 1,3,5-triazines derivatives containing aminopyridine or thiopyridine, it has been demonstrated their ability to induce apoptosis by arresting G2/M phase of cell cycle and involvement of protein p53 [30].

According to the preliminary results, novel 1,3,5-triazine derivatives, which contain one, two or three 2- chloroethylamine fragments showed anti-proliferative activity against breast cancer T47D, colorectal cancer SW707, prostate cancer LNCaP, lung cancer A549 and Jurkat lymphoblastic leukemia cells. Moreover, their efficiency has been increased with the number of 2- chloroethylamine moieties. In contrast the breast cancer MCF-7 cell line was resistant to this structural modification [31].

The recent study focused on synthesis of aryloamino-1,3,5-triazines functionalized with alkylating 2-chloroethylamine fragments, where alkoxy substituent was replaced on the triazine ring with alkyl-(aryl-) amine group. The obtained compounds were tested for in vitro antiproliferative activity, using the standard human MDA-MB-231 and MCF-7 breast cell lines, and were found that they induce necrosis less intensely that apoptosis. The inhibitory activity in case of MCF-7 cells was found strongly dependent on the structure of substituents on the triazine ring. In turn, the amount number of alkylating fragments was not significant for activity [32].

**Fig. 1:**
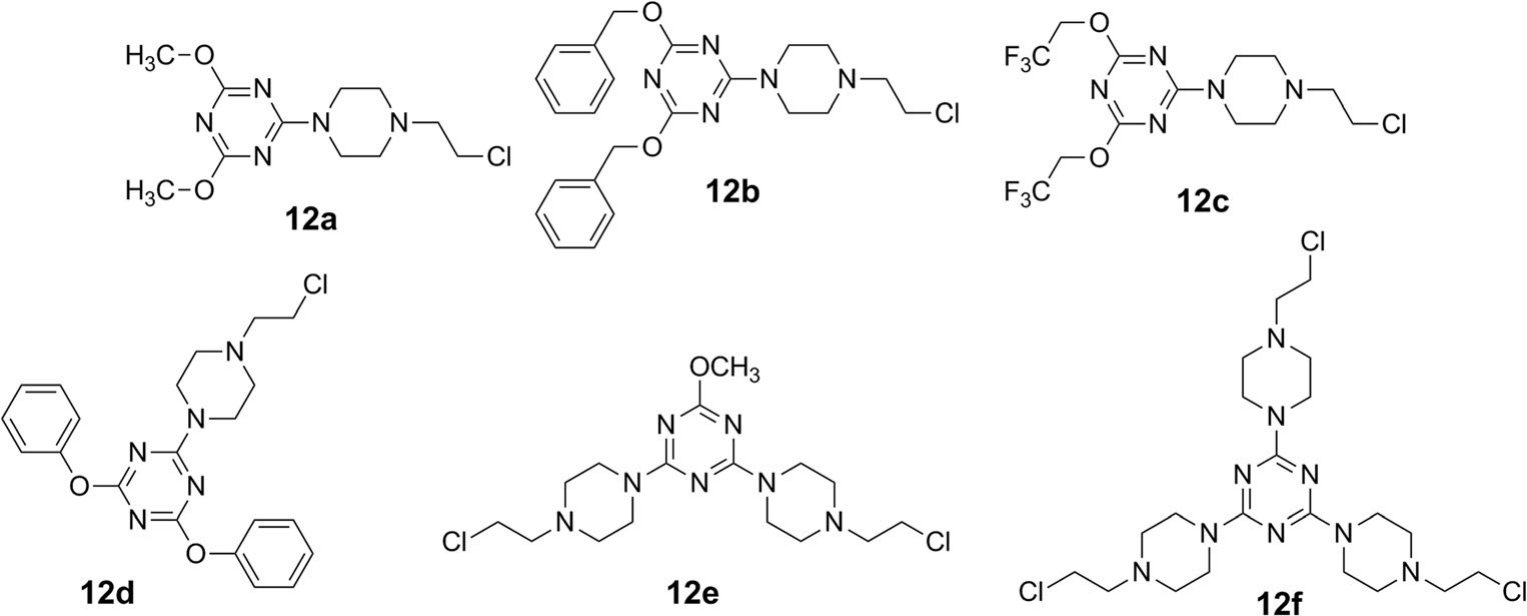
Structures of triazine mustards

These studies provide strong evidence that tiny structure modification of alkylating agents, including nitrogen mustards, can determine in vitro the profile of anti-cancer activity. In this report, we made an effort to extend the range of knowledge about antitumor activity of 1,3,5-triazines. To reach this goal it has been investigated the influence of previously synthesized 1,3,5-triazines derivatives bearing one- (**12a-d**), two- (**12e**) and three- (**12f**) 2-chloroethyloamino residues (Fig. 1) on LBC3, LN-18 and LN-229 glioblastoma cell lines in order to determine the cell type-specific effects.

## Materials and methods

### Reagents

The DMEM with GlutaMax™, trypsin-EDTA, penicillin, streptomycin, FBS Gold were provided by Invitrogen (*San Diego, CA, USA*) and Annexin V Apoptosis Detection Kit I by BD Pharmingen™ (*San Jose, CA, USA*). The DMSO, acridine orange, bromide ethidium and 3-(4,5-dimethylthiazol-2-yl)-2,5-diphenyltetrazolium bromide by Sigma (*St Louis, MO, USA*). Triazine mustards **12a-d** were obtained according to standard procedure described earlier [31].

### Cell cultures

The LBC3 cell line was developed from *GMB* tissue taken from 56-year-old female patient subjected to surgical tumour resection, and was kindly given to us by Prof. *Cezary Marcinkiewicz* (Department of Neuroscience, Temple University, Philadelphia, PA, USA) [33]. LN-18 and LN-229 cells were purchased from American Type Culture Collection (ATCC). The cells were cultured in Dulbecco’s modified Eagle’s medium (DMEM) with GlutaMax™, supplemented with heat-inactivated, 10% fetal bovine serum GOLD (FBS GOLD), streptomycin (100 μg/mL) and penicillin (100 U/mL). The cells were cultured in Falcon flasks (BD) at 37 °C in a humidified atmosphere of 5% CO_2_, 95% air in incubator Galaxy S+ (RS Biotech). At approximately 70% confluence, cells were detached with 0.05% trypsin, 0.02% EDTA in calcium-free phosphate-buffered saline and counted in a Scepter cell counter (Millipore). Then, 2.0 × 10^5^ cells were seeded in 2 mL of growth medium in 6-well plates or 2.0 × 10^4^ cells were seeded in 1 mL of growth medium in 24-well plates. After 24 h the medium was removed and replaced with fresh medium containing: **12a-f** or **CHL** at concentrations from 1 μM to 150 μM. The glioma cells not treated with compounds or **CHL** served as the negative control.

Normal primary skin fibroblasts (p8) were purchased from ATCC (CRL1474). Fibroblast cultures were passaged in high glucose (4.5 g/L) DMEM without L-glutamine, with 10% FBS in six-well plates (Falcon). Cultures were grown at 37 °C in an atmosphere containing 5% CO_2_.

### Cell viability

Cell viability was measured according to the method of Carmichael [34] using 3-(4,5-dimethylthiazol-2-yl)-2,5-diphenyltetrazolium bromide (MTT). Briefly, cells were seeded in 24-well plate at a density of 2.0 × 10^4^ per well. After 24 h the medium was removed and replaced with fresh medium containing **12a-f** or **CHL**, at concentrations from 1 μM to 150 μM. The LN-18, LN-229 or LBC3 cells not treated with compounds served as negative control. Next, the cells were incubated for 24 and 48 h and washed three times with PBS and then incubated with 1 mL of MTT solution (0.25 mg/mL in PBS) for 4 h at 37 °C in 5% CO_2_ in an incubator. The medium was removed and 1 mL of 0.1 mol/l HCl in absolute isopropanol was added. Absorbance of converted dye in living cells was measured at the wavelength of 570 nm. The viability of cells cultured with **12a-f** compounds were calculated as the percentage of control cells incubated without compounds or **CHL**. The viability of the cells were analyzed and IC_50_ was calculated.

### Detection of apoptosis and necrosis

Apoptosis and necrosis of LBC3, LN-18 or LN-229 human glioblastoma cells were evaluated by flow cytometry on FACSCanto II cytometer (Becton Dickinson). The cells were seeded in 6-well plate at a density of 2.0 × 10^5^ per well. After 24 h the medium was removed and replaced with fresh medium containing **12a-f** or **CHL**, at concentrations from 1 μM to 150 μM. The LBC3, LN-18 or LN-229 cells not treated with compounds served as the negative control. Next, the cells were trypsinized, resuspended in DMEM and then in binding buffer. Subsequently cells were stained with FITC Annexin V and PI for 15 min at room temperature, in the dark, following the manufacturer’s instructions (FITC Annexin V apoptosis detection Kit I). Data were analyzed by use of FACSDiva software and dead cells were excluded based on forward- and side-scatter parameters. Percentage of apoptotic cells was presented as a sum of Q2 and Q4 quadrant, necrotic cells as a Q1 quadrant population of analyzed cells.

### Cell morphological analysis

Staining cells with fluorescent dyes, including acridine orange and ethidium bromide, were used in evaluation of the nuclear morphology of apoptotic and necrotic cells. The glioma LBC3, LN-18 and LN-229 cells grown with compounds for 24 and 48 h. Next, the cells were washed twice with PBS and stained with 1 mL of the dyes mixture (10 μM acridine orange and 10 μM ethidium bromide in PBS) at room temperature for 10 min in dark. Next, stained solution was removed, the cells layer were washed with PBS, analyzed and photographed under a fluorescence microscope (Olympus CXK41, U-RLFT50) at 200-fold magnification according to the following criteria: normal green nucleus – living cells; orange or green stained nuclei with chromatin condensation or fragmentation – apoptotic cells, while necrotic cells were characterized by orange stained cell nuclei.

### Statistical analysis

Mean values for seven assays ± standard deviations (SD) were calculated. Statistical analysis was performed using Student’s *t* test.

## Results

### The effect of compounds or CHL on cell viability

**Fig. 2:**
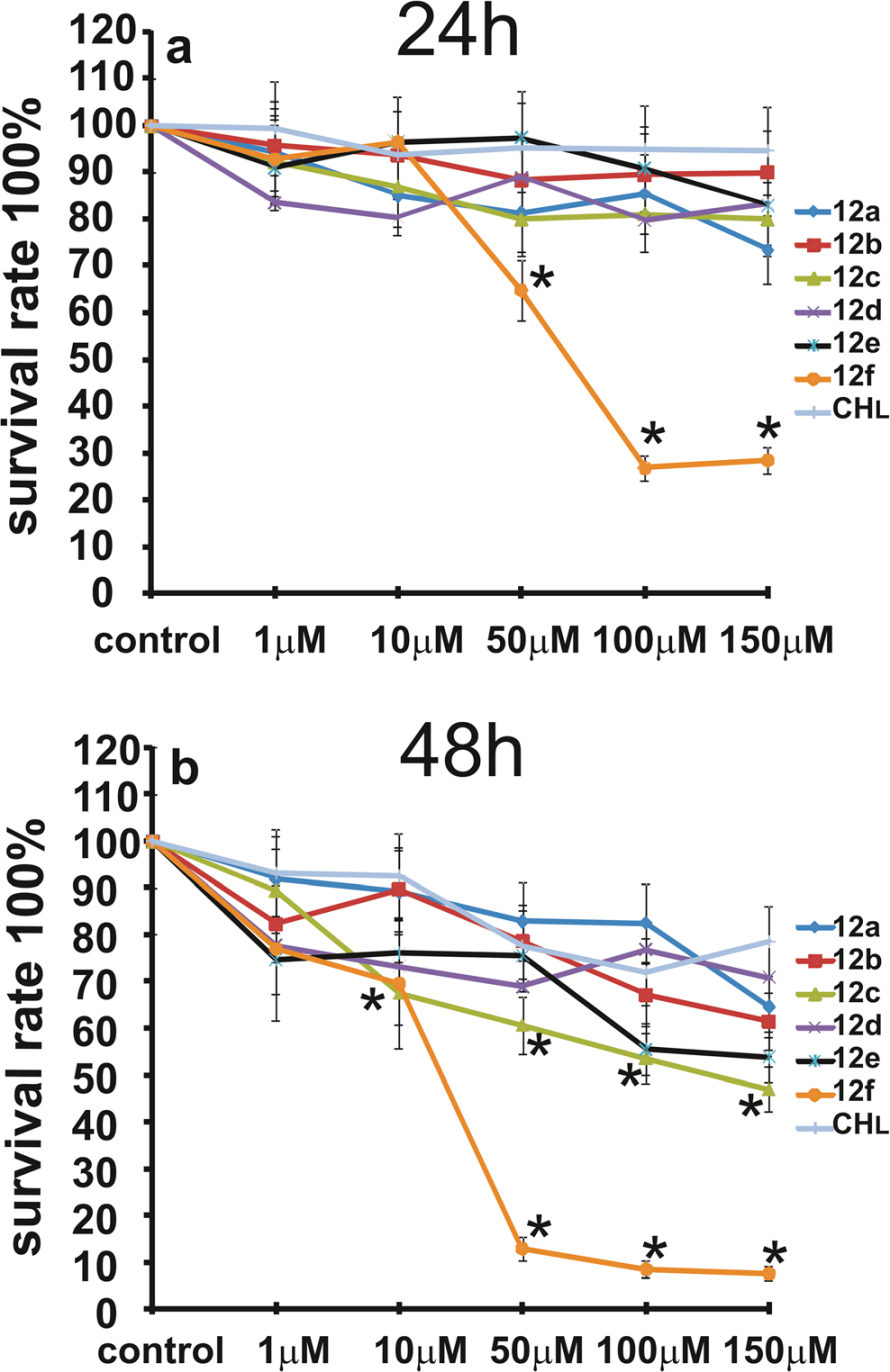
The viability of LBC3 cells treated with different concentrations of compounds **12a**-**f** or **CHL**, from 1 to 150 μM for 24 (**a**) and 48 h (**b**). Mean values from three independent experiments ± SD are presented. Significant alterations are expressed relative to controls and marked with asterisks. Statistical significance was considered if * *p* < 0.05

**Fig. 3:**
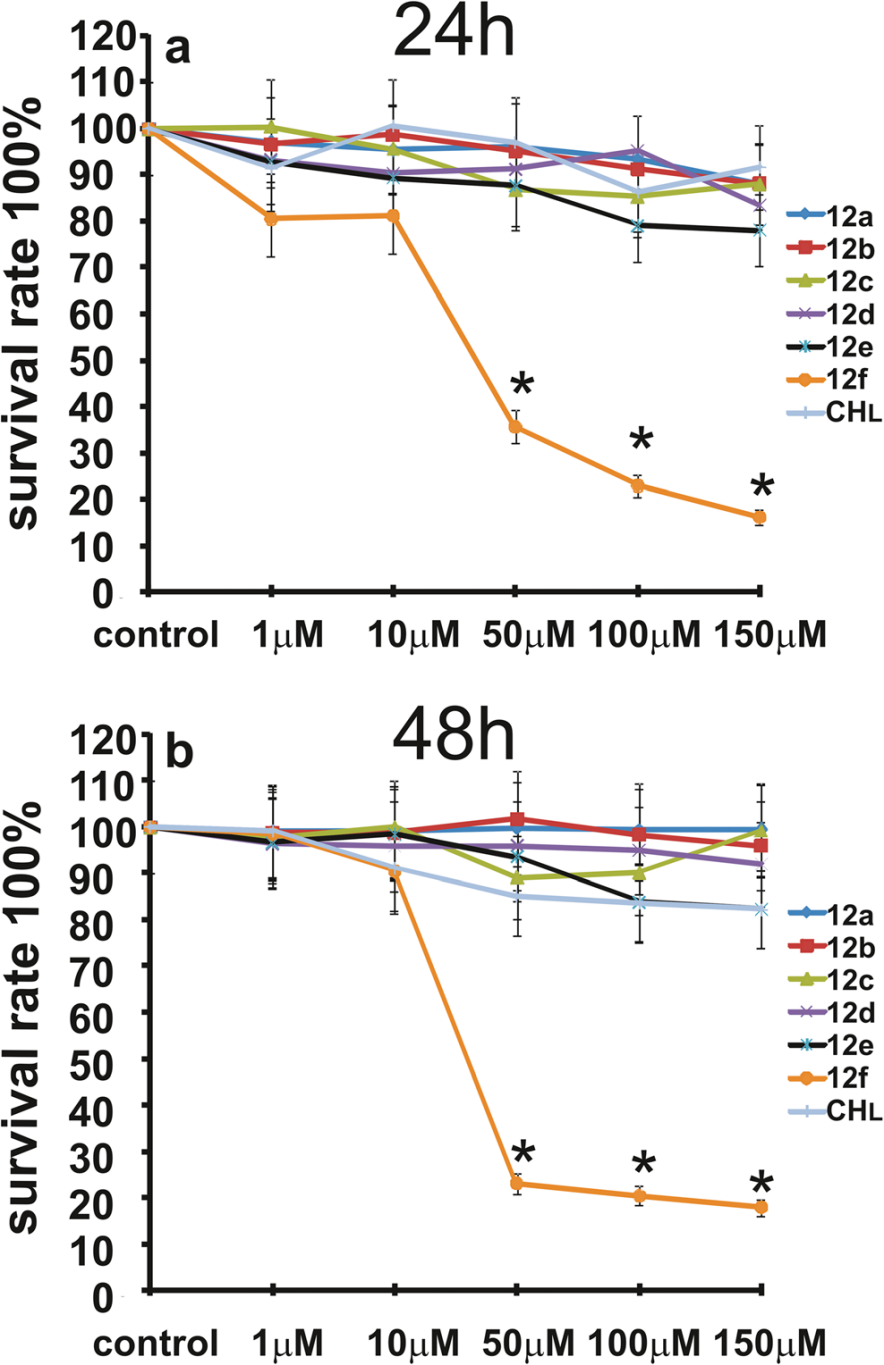
The viability of LN-18 cells treated with different concentrations of compounds **12a**-**f** or **CHL**, from 1 to 150 μM for 24 (**a**) and 48 h (**b**). Mean values from three independent experiments ± SD are presented. Significant alterations are expressed relative to controls and marked with asterisks. Statistical significance was considered if *p < 0.05

**Fig. 4:**
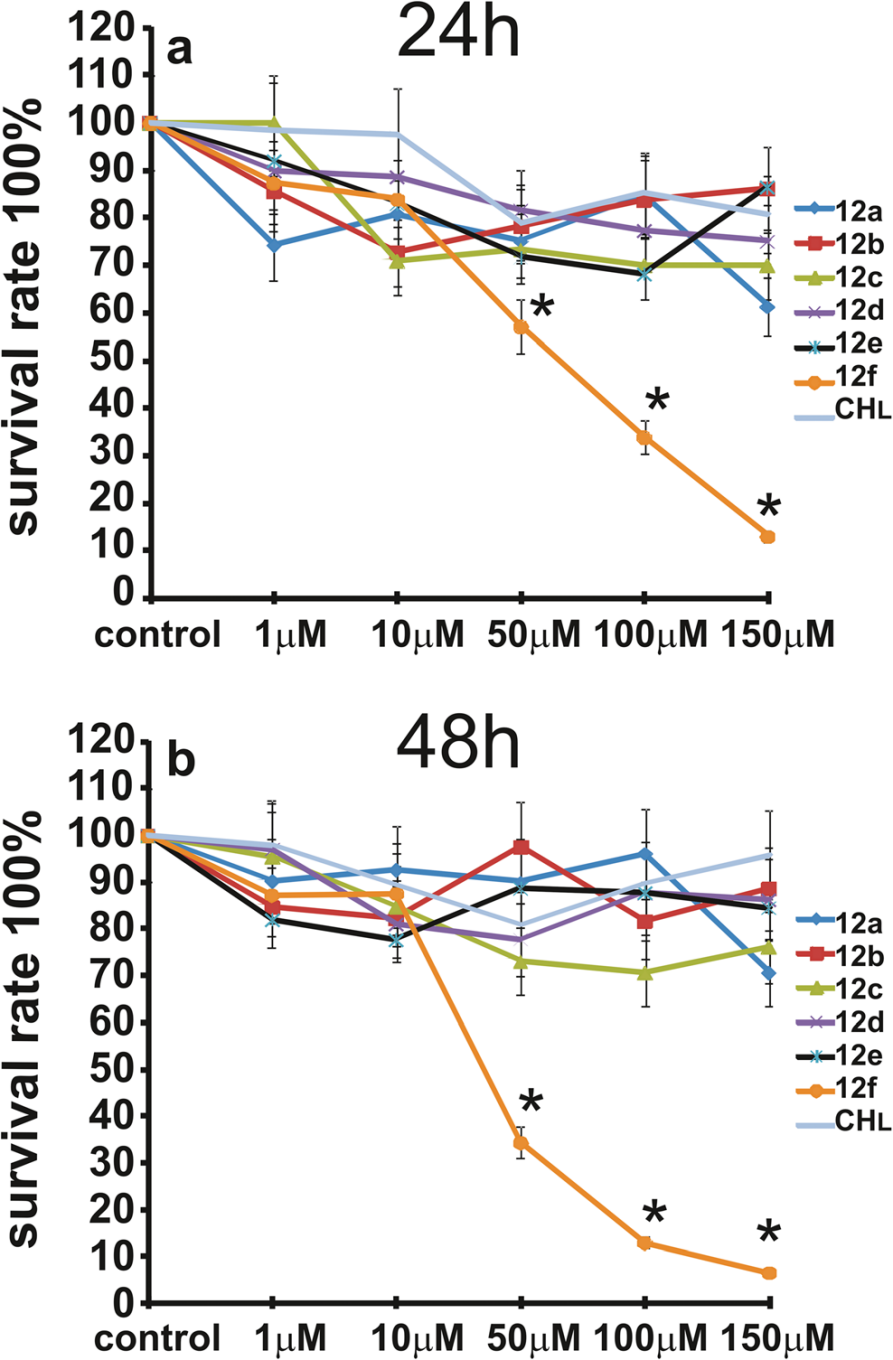
The viability of LN-229 cells treated with different concentrations of compounds **12a**-**f** or **CHL**, from 1 to 150 μM for 24 (**a**) and 48 h (**b**). Mean values from three independent experiments ± SD are presented. Significant alterations are expressed relative to controls and marked with asterisks. Statistical significance was considered if *p < 0.05

The anti-proliferative effects of compounds, **12a-f** or chlorambucil (**CHL**) in glioblastoma: LBC3 (Fig. 2), LN-18 (Fig. 3) as well as LN-229 (Fig. 4) cells, were studied by MTT 3-(4,5-dimethylthiazol-2-yl)-2,5-diphenyltetrazolium bromide assay. The cell lines were incubated with increasing concentrations of **12a-f** compounds or **CHL**, ranging from 1 μM to 150 μM, for 24 and 48 h. Figs. 2, 3 and 4 indicate that all compounds or CHL caused time-dependent and dose-dependent reduction in viability of all three tested glioblastoma cells. However, the cytotoxic effect was dependent on kind of compounds. The biggest reduction in cell viability of LBC3, LN-18 and LN-229 cells was observed after 24 and 48 h of incubation with **12f** compound with concentrations ranging from 50 μM to 150 μM. In these cells treated with higher concentrations of **12f**, the effect on cell viability was clearly the strongest of all examined compounds (Figs. 2, 3, 4). Interestingly, in all tested cell lines, exposed to the highest concentration (150 μM) of **12f,** after 24 h we observed reduced cell viability approximately to 71% in LBC3; 84% in LN-18 and 87% in LN-229 in comparison to the control cells (Figs. 2a, 3a and 4a). After 48 h of treatment, the viability of cells exposed to the highest concentration (150 μM) of **12f**, was reduced by 93% in LBC3 and LN-229 and cells was comparable to viability after 24 h in LN-18 (Figs. 2b, 3b and 4b). This phenomenon was substantially more significant compared to the other compounds studied. None of the tested compounds had an anti-proliferative effect on fibroblast cells (data not shown).

The half maximal inhibitory concentration (IC_50_) values of compound **12f** was calculated using GraphPad Prims 5. Our results demonstrated that after 24 h of incubation IC_50_ of compound **12f** was: 49 ± 3 μM, 60 ± 2 μM, 52 ± 3 μM for LN-18, LN-229 or LBC3 cell lines, respectively. After 48 h of incubation IC_50_ of compound **12f** was: 46 ± 2 μM, 50 ± 2 μM and 40 ± 3 μM for LBC3, LN-18 and LN-229, respectively.

### The effect of compounds or CHL on apoptosis and necrosis

**Fig. 5:**
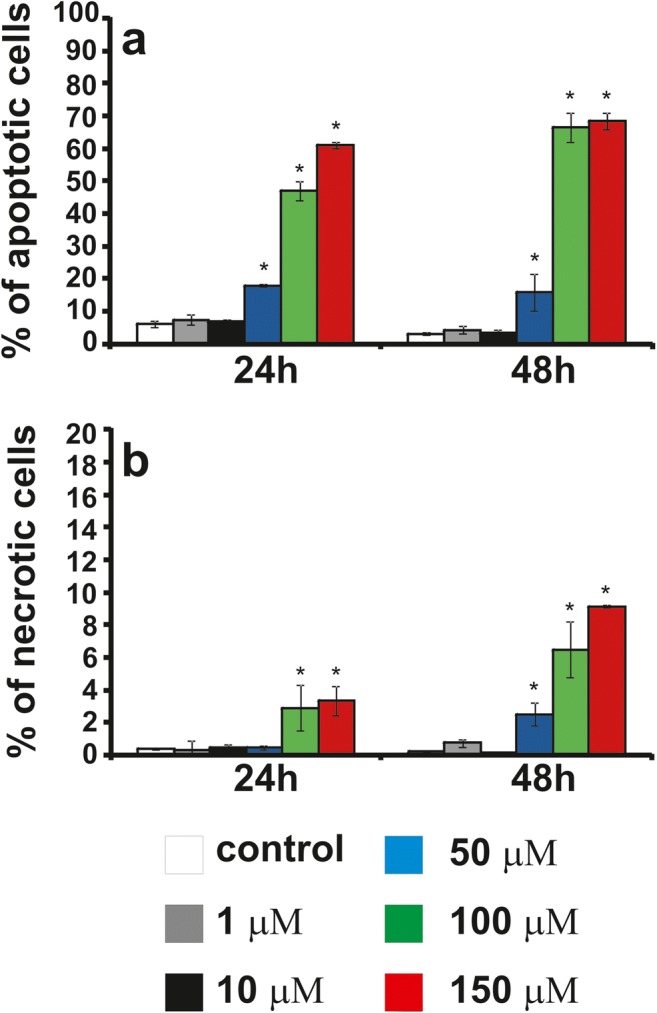
The effect of **12f**, from 1 μM to 150 μM, on percentage of apoptotic (**a**) and necrotic cells (**b**) of glioblastoma LBC3 cell line incubated for 24 and 48 h. Mean values from three independent experiments ± SD are presented. Significant alterations are expressed relative to adequate controls and marked with asterisks; *p < 0.05

**Fig. 6:**
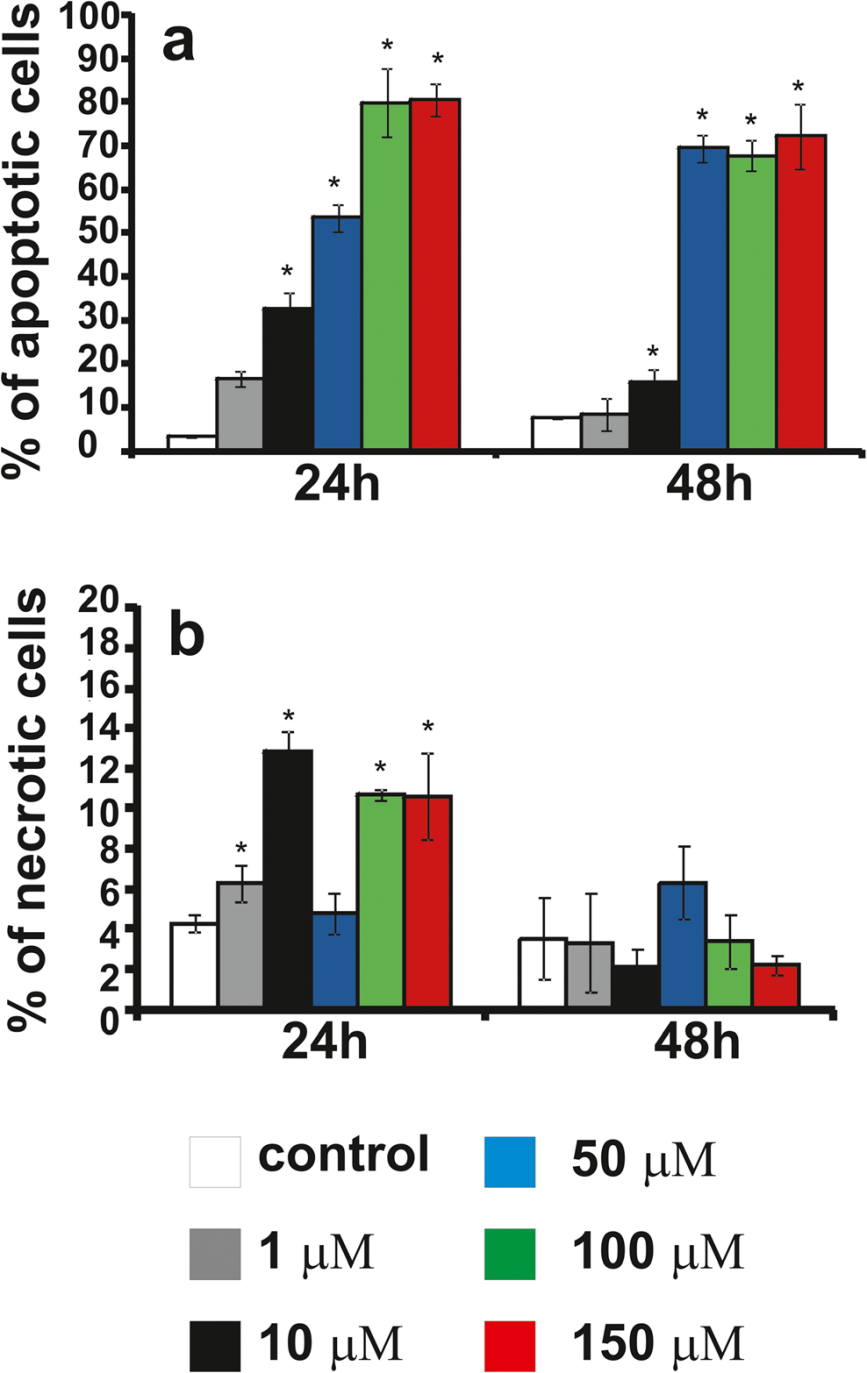
The effect of **12f**, from 1 μM to 150 μM, on percentage of apoptotic (**a**) and necrotic cells (**b**) of glioblastoma LN-18 cell line incubated for 24 and 48 h. Mean values from three independent experiments ± SD are presented. Significant alterations are expressed relative to adequate controls and marked with asterisks; *p < 0.05

**Fig. 7:**
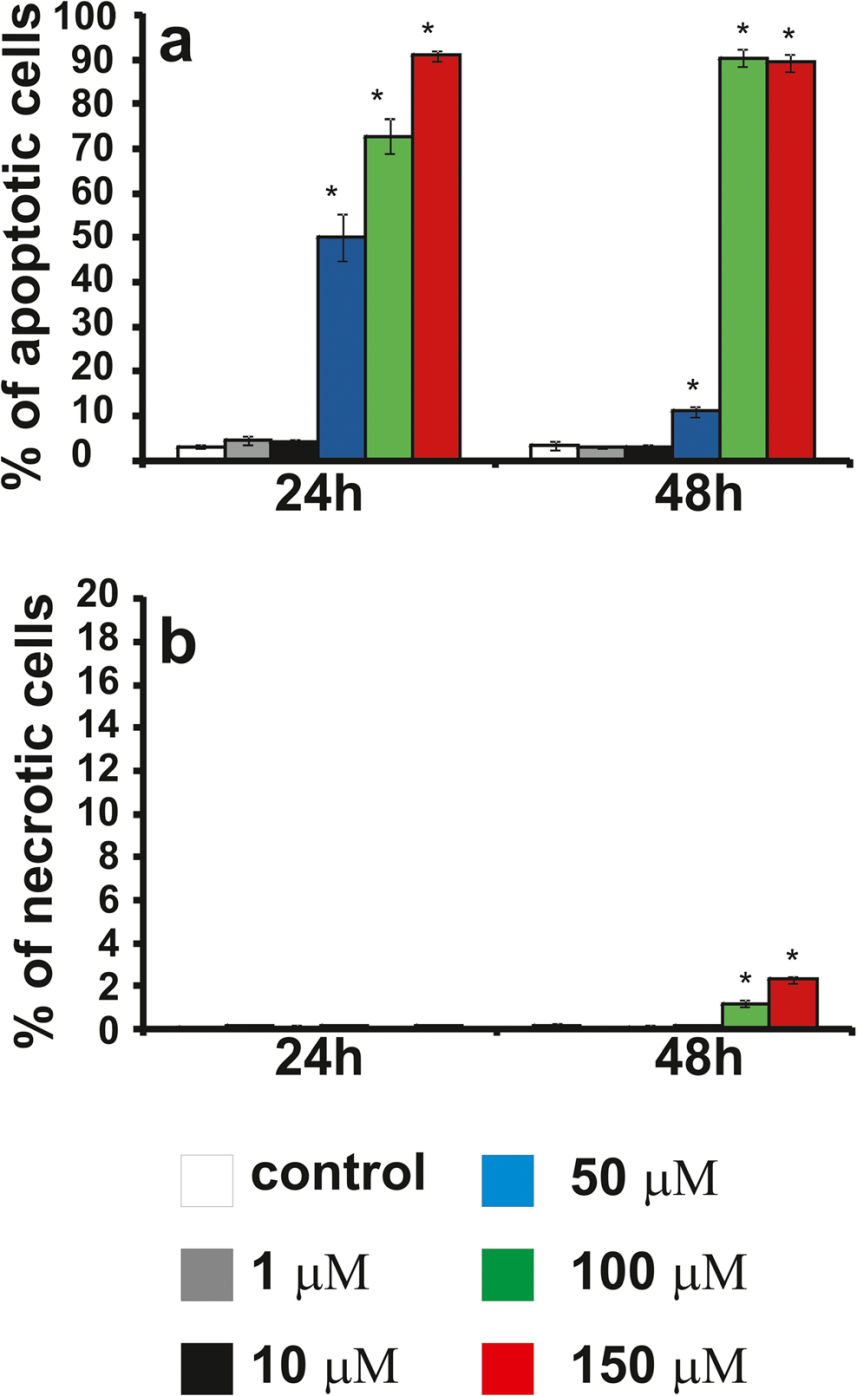
The effect of **12f**, from 1 μM to 150 μM, on percentage of apoptotic (**a**) and necrotic cells (**b**) of glioblastoma LN-229 cell line incubated for 24 and 48 h. Mean values from three independent experiments ± SD are presented. Significant alterations are expressed relative to adequate controls and marked with asterisks; *p < 0.05

We investigated whether **12f** toxicity was due to the induction of apoptosis. The LBC3 (Fig. 5), LN-18 (Fig. 6) and LN-229 (Fig. 7) cells were incubated with concentrations of **12f** increased gradually from 1 μM to 150 μM, for 24 and 48 h. In the all investigated cells, incubated for 24 and 48 h in the medium with **12f** compound, in the concentration ranging from 50 μM to 150 μM, we observed a time- and dose-dependent increase in apoptosis in comparison to the control cells (Fig. 5a, Fig. 6a, Fig. 7a). Interesting is the fact, that the amount of apoptotic cells was dependent on investigated cell line. As depicted in Fig. 5a the percent of apoptotic LBC3 cells, incubated with **12f** compound with concentrations ranging from 50 μM to 150 μM, was ranging from 20 to 70%, in LN-18 cells from 50 to 90% (Fig. 6a) and in LN-229 from 10 to 90% (Fig. 7a).

Next our research has shown slight necrosis in the case of all three cell lines LBC3 (Fig. 5b), LN-18 (Fig. 6b) and LN-229 (Fig. 7b) incubated for 24 and 48 h in medium with compound **12f** in concentrations from 1 μM to 150 μM.

### Cell morphological analysis

**Fig. 8:**
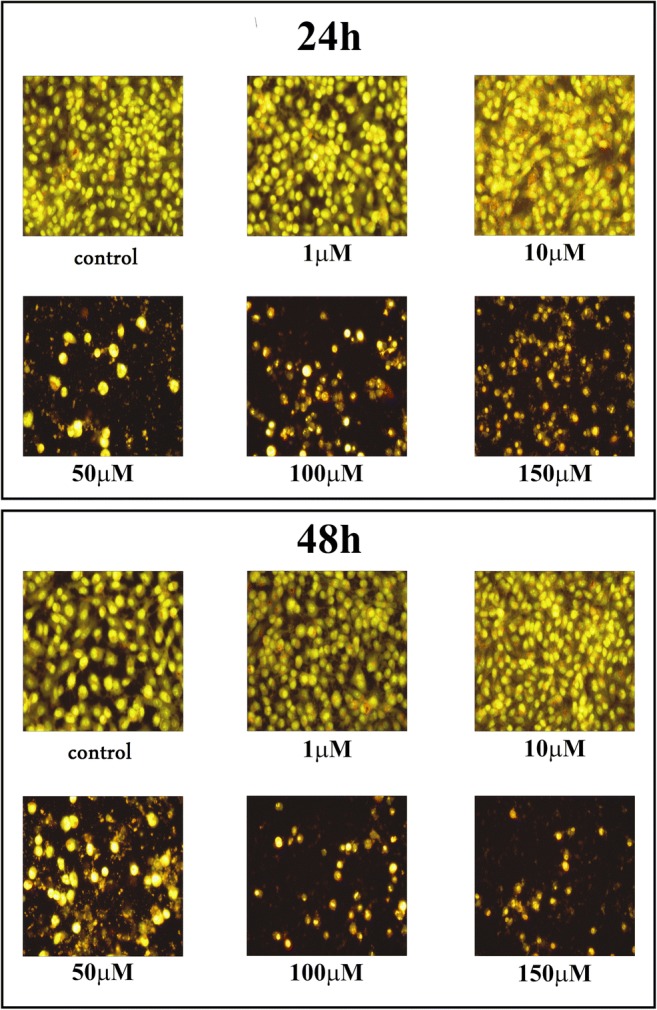
The effect of **12f** on apoptosis, necrosis in LBC3 cells evaluated by fluorescence microscope assay. The cells were incubated in medium with different concentrations of compound **12f** for 24 and 48 h and stained with acridine orange and ethidium bromide. The cells were photographed under a fluorescence microscope at 200-fold magnification and analyzed according to the following criteria: living cells, apoptotic cells, and necrotic cells. We presented representative images form one of three independent experiments

**Fig. 9:**
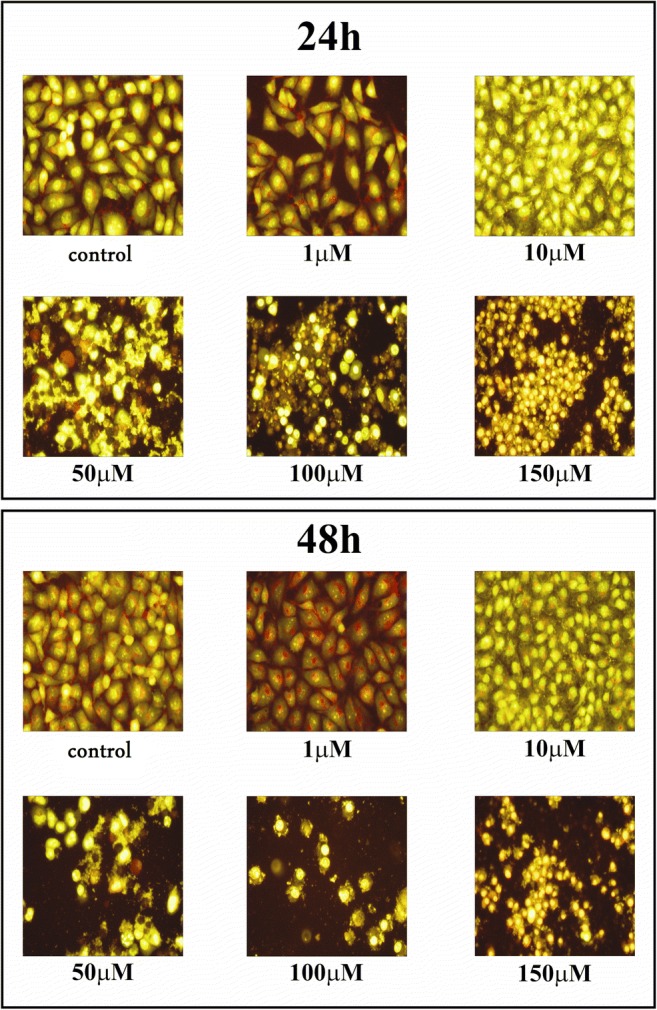
The effect of **12f** on apoptosis, necrosis in the LN-18 cells evaluated by fluorescence microscope assay. The cells were incubated in medium with different concentrations of compound **12f** for 24 and 48 h and stained with acridine orange and ethidium bromide. The cells were photographed under a fluorescence microscope at 200-fold magnification and analyzed according to the following criteria: living cells, apoptotic cells, and necrotic cells. We presented representative images form one of three independent experiments

**Fig. 10:**
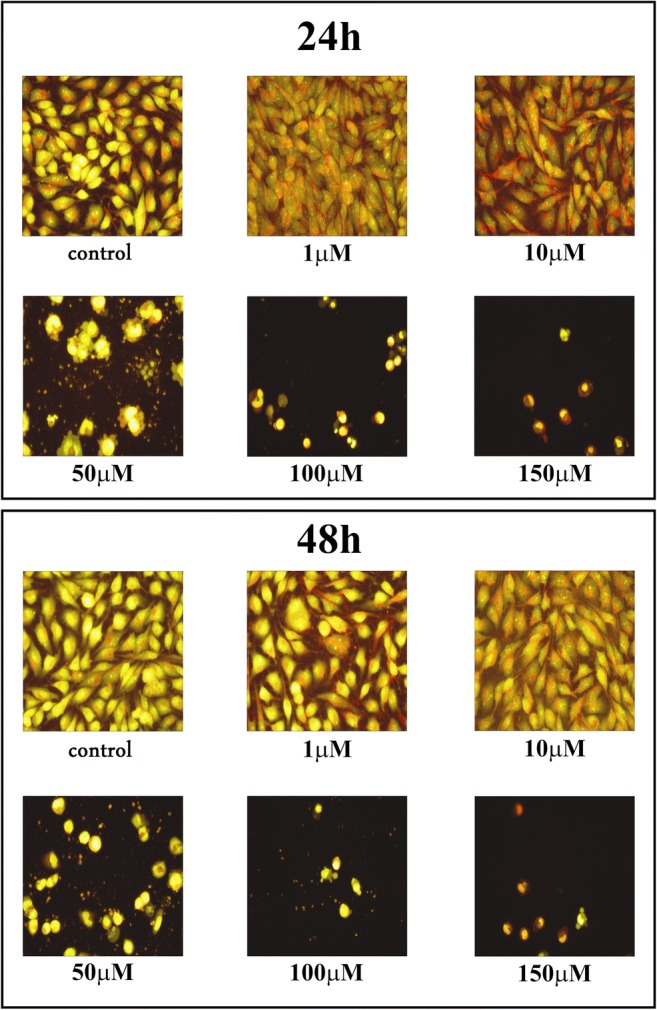
The effect of **12f** on apoptosis, necrosis in the LN-229 cells evaluated by fluorescence microscope assay. The cells were incubated in medium with different concentrations of compound **12f** for 24 and 48 h and stained with acridine orange and ethidium bromide. The cells were photographed under a fluorescence microscope at 200-fold magnification and analyzed according to the following criteria: living cells, apoptotic cells, and necrotic cells. We presented representative images form one of three independent experiments

Staining of the cells with fluorescent dyes, including acridine orange and ethidium bromide, was used for the evaluation of apoptotic and necrotic cells morphology under fluorescent microscope. The method distinguished viable cells by appearing with green nuclei without condensation of chromatin, apoptotic cells by showing green or red nuclei with condensation of chromatin, whiles the necrotic cells - by red nuclei without condensation of chromatin. The figures: 8, 9, 10 show that **12f** compound caused reduction of cells viability in time- and dose-dependent manner. We did not observe significant changes in nuclear morphology between the cells incubated with 1 μM or 10 μM of **12f** compound, for 24 h, in comparison to the control cells. However, in case of the cells incubated with compound **12f** at concentration ranging from 50 μM to 150 μM, we noticed the apoptotic cells with condensed, fragmented and marginalized chromatin, nuclear shrinking, when compared with controls. Furthermore, the apoptotic bodies and shrinkage of the cells were observed. Otherwise, after 24 h incubation with 100 μM or 150 μM of compound **12f**, we noticed necrotic cells with red nuclei without condensation of chromatin. The cells incubated for 48 h with different concentrations of **12f** compound were characterized as more red-stained, with condensation of chromatin, indicative the late apoptosis and strong increased shrinkage of the cells and number of apoptotic bodies. Apart from that, the number of necrotic cells was lower than the number of apoptotic cells after 24 as well as 48 h. We did not observed changes in the morphology of nuclei between the analyzed glioblastoma cell lines: LBC3 (Fig. 8), LN-18 (Fig. 9), LN-229 (Fig. 10).

## Discussion

Glioblastoma multiforme is one of the most commonly occuring malignant types of primary brain tumors [35]. These include: astrocytic tumors (astrocytoma, anaplastic astrocytoma and glioblastoma), oligodendrogliomas, ependymomas and mixed gliomas. Increase in number of diagnosed brain tumors leads to the search for new therapeutic strategies [36].

The 1,3,5-triazines core is versatile scaffold for mono, two or three 2-chloroethylamine groups. The modification of 1,3,5-triazine ring system can lead to numerous novel and diverse target-specie inhibitors of enzymes important for the proper functioning of cells [31]. Furthermore, 1,3,5-triazines have been widely studied due to it is broad range of biological activities including their anticancer or antimicrobial effects [37]. 1,3,5-triazines don’t act selectivity, causing high levels of inadvertent DNA damage structure and apoptosis in not cancer cells. For this reason, the search for the new analogues is continued expecting improvement of therapeutic index by modification of triazines molecular structure for example by addition of 2-chloroethylamine groups [32].

Chemotherapeutic agents can be classified as cytotoxic or cytostatic. Cytotoxic agents lead to cancer cell death. Several mechanisms of action are common among the cytotoxic agents used for glioma cells, including DNA alkylation, DNA cross-linkage, DNA breaks, whiles cytostatic agents alter tumor biology by inhibiting tumor growth [39]. It was found correlation between the alkylating activity of the 1,3,5-triazines with 2-chloroethylamine groups and their cytotoxicity on the breast cancer MCF-7 cell line. In addition it was indicated that alkylating activity increase with the number of 2-chloroethylamino groups [31]. It is interesting, that 1,3,5-triazine can modulate the functions: HIV-1 reverse transcriptase, estrogen receptor β (ΕRβ), glutathione S-transferase [38, 40]. 1,3,5-triazines with 2-chloroethylamino fragment can inhibit DNA replication, cell cycle arrest, induce of apoptosis and inhibit of cancer cells growth [31]. For many cell lines: breast cancer (T47D), prostate cancer (LNCaP), colorectal cancer (SW707), lung cancer (A549) and Jurkat lymphoblastic leukemia, the strong inhibition of viability was observed for compounds **12a-f** or **CHL** [2]. In contrast, our research showed that glioma LBC3, LN-18 and LN-229 cell lines are resistant to compounds **12a-12e** and **CHL**, while their viability was strong decreased only by 1,3,5-triazine **12f** substituted with three 2-chloroethylamine groups. We suggest, that **12f** compound leads to alkylation of DNA and decreases of cell viability in glioblastoma LBC3, LN-18 and LN-229 cell lines as well as can lead to their apoptosis. This characteristic types of cell death, are discussed in our previous paper [35]. Program cell death (PCD), also termed type 1 PCD, plays an important role in development of tissue homeostasis, degenerative diseases and cancer [40]. Apoptosis is a complex and multi-stage process. During this process a variety of biochemical and morphological changes take place through different signal transduction pathways [41]. The two main mechanisms of the induction of apoptosis, the mitochondrial (intrinsic) pathway and receptor-mediated (extrinsic) pathway, are commonly known [42]. In our study, we indicated the apoptosis and necrosis occurring in LBC3, LN-18 and LN-229 cells after stimulation with the compound **12f**. The effect of **12f** on the apoptosis and necrosis was investigated using the Annexin V-FITC and propidium iodide (PI) followed by biparametric flow cytometry analysis. Mono-functional analogues of 1,3,5-triazines, which are unable to form liaisons between two DNA strands, were also found to induce apoptosis and necrosis [31, 43]. New compounds **12a-f** induced dose-dependent apoptosis and necrosis of MCF-7 cell line [31]. Chromatin condensation and DNA fragmentation are one of the most crucial criteria, which are used to identify apoptotic cells [42]. In this study we observed chromatin fragmentation, condensation, and apoptotic bodies in LN-18, LBC3 and LN-229 cell lines. During apoptosis, chromatin undergoes a phase change from a heterogeneous, genetically active network to an inert, highly condensed form [42].

Apart from that, proapoptotic effect on glioblastoma LN-18, LBC3 and LN-229 cell lines were found to be strongly dependent on the structure of the substituents on the 1,3,5-triazine ring.

**Fig. 11:**
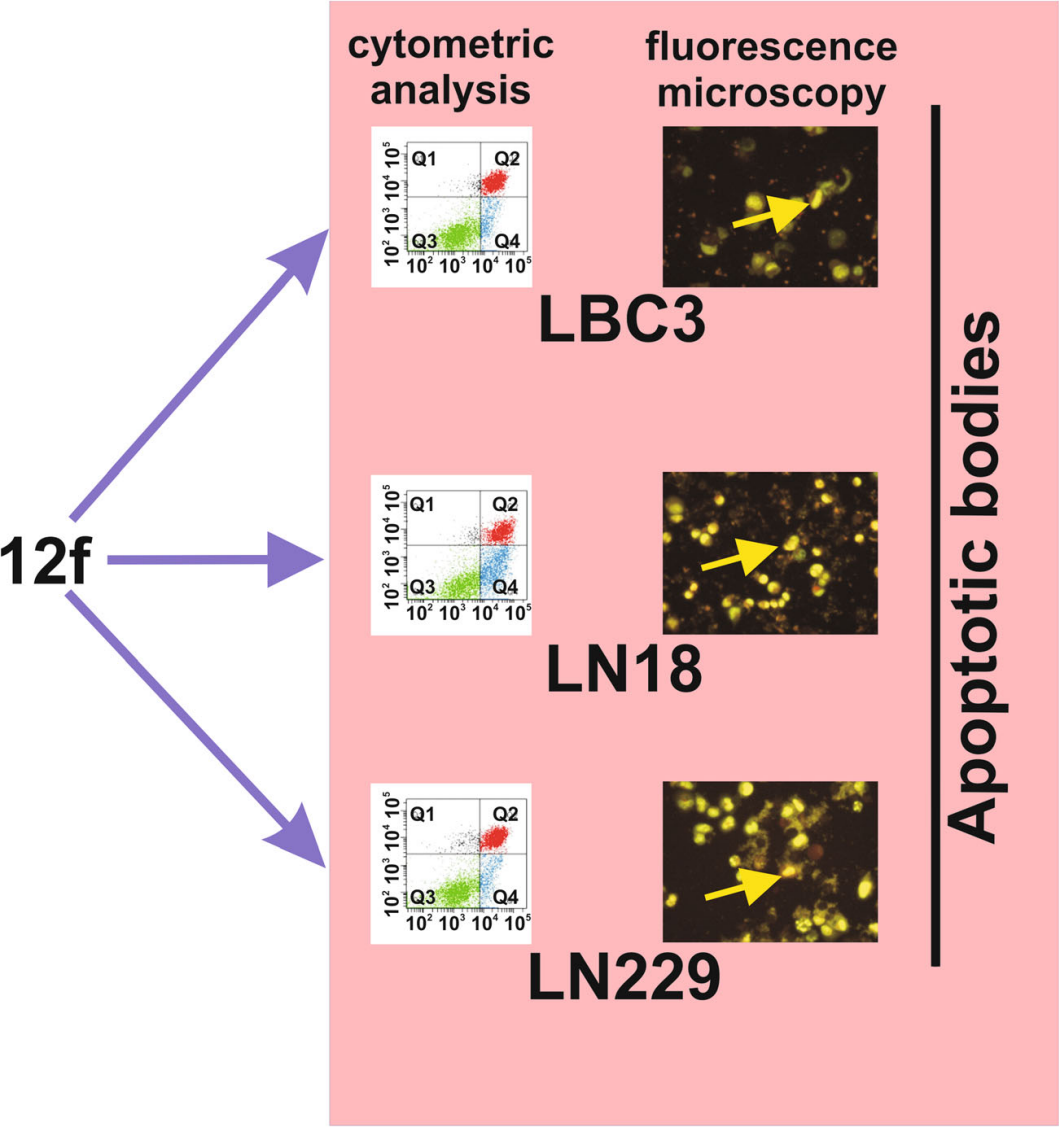
The effect of compound **12f** on apoptosis and necrosis in glioma LBC3, LN-18 and LN-229 cell lines

In conclusion, based on the present data, the new compound **12f,** which is able to induce strong apoptosis in investigated glioma cells (Fig. 11), may be a good candidate for further evaluation as an effective chemotherapeutic agent for human glioblastoma cells.

## References

[1] Wen PY, Kesari S (2008). Malignant gliomas in adults. N Engl J Med.

[2] Amberger-Murphy V (2009). Hypoxia helps glioma to fight therapy. Curr Cancer Drug Targets.

[3] JJr P, Polivka J, Rohan V, Topolcan O, Ferda J (2012). New molecularly targeted therapies for glioblastoma multiforme. Anticancer Res.

[4] Burton EC, Lamborn KR, Forsyth P, Scott J, O’Campo J, Uyehara-Lock J, Prados M, Berger M, Passe S, Uhm J, O'Neill BP, Jenkins RB, Aldape KD (2002). Aberrant p53, mdm2, and proliferation differ in glioblastomas from long-term compared with typical survivors. Clin Cancer Res.

[5] Omuro A, DeAngelis LM (2013). Glioblastoma and other malignant gliomas: a clinical review. JAMA.

[6] Tomicic MT, Meise R, Aasland D, Berte N, Kitzinger R, Krämer OH, Kaina B, Christmann M (2015). Apoptosis induced by temozolomide and nimustine in glioblastoma cells is supported by JNKc-Jun-mediated induction of the BH3-only protein BIM. Oncotarget.

[7] Friedman HS, Kerby T, Calvert H (2000). Temozolomide and treatment of malignant glioma. Clin Cancer Res.

[8] Marosi C, Bogdahn U, Curschmann J, Janzer RC, Ludwin SK (2005). Radiotherapy plus concomitant and adjuvant temozolomide for glioblastoma. N Engl J Med.

[9] Johnson DR, O’Neill BP (2012). Glioblastoma survival in the United States before and during the temozolomide era. J Neuro-Oncol.

[10] Shahar T, Nossek E, Steinberg DM, Rozovski U, Blumenthal DT, Bokstein F, Sitt R, Freedman S, Corn BW, Kanner AA, Ram Z (2012). The impact of enrolment in clinical trials on survival of patients with glioblastoma. J Clin Neurosci.

[11] Tong WP, Kirk MC, Ludlum DB (1982) Formation of the crosslink 1-[N3-deoxycytidyl), 2[N1-deoxyguanosinyl]-ethane in DNA treated with N,N′-bis(2-chloroethyl)-N-nitrosourea. Cancer Res 42:3102–3105 7093954

[12] Ludlum DB (1997). The chloroethyl nitrosoureas: sensitivity and resistance to cancer chemotherapy at the molecular level. Cancer Investig.

[13] Fischhaber PL, Gall AS, Duncan JA, Hopkins PB (1999). Direct demonstration in synthetic oligonucleotides that N,N′-bis(2-chloroethyl)-nitrosourea cross links N1 of deoxyguanosine to N3 of deoxycytidine on opposite strands of duplex DNA. Cancer Res.

[14] Giese A, Bjerkvig R, Berens ME, Westphal M (2003). Cost of migration: invasion of malignant gliomas and implications for treatment. J Clin Oncol.

[15] Svechnikova I, Almqvist PM, Ekström TJ (2008). HDAC inhibitors effectively induce cell type-specific differentiation in human glioblastoma cell lines of different origin. Int J Oncol.

[16] Hegi ME, Liu L, Herman JG, Stupp R, Wick W, Weller W, Mehta MP, Gilbert MR (2008). Correlation of O6-methyl guanine methyltransferase (MGMT) promoter methylation with clinical outcomes in glioblastoma and clinical strategies to modulate MGMT activity. J Clin Oncol.

[17] Becker T, Gerke V, Kube E, Weber K (1992). S100P, a novel Ca(2+)-binding protein from human placenta. cDNA cloning, recombinant protein expression and Ca2+ binding properties. Eur J Biochem.

[18] Sims JN, Graham B, Pacurari M, Leggett SS, Tchounwou PB, Ndebele K (2014). Di-ethylhexylphthalate (DEHP) modulates cell invasion, migration and anchorage independent growth through targeting S100P in LN-229 glioblastoma cells. Int J Environ Res Public Health.

[19] Nielsen JS, McNagny KM (2009). The role of podocalyxin in health and disease. J Am Soc Nephrol.

[20] Wu H, Yang L, Liao D, Chen Y, Wang W, Fang J (2013). Podocalyxin regulates astrocytoma cell invasion and survival against temozolomide. Exp Ther Med.

[21] Kaprio T, Fermér C, Hagström J, Mustonen H, Böckelman C, Nilsson O, Haglund C (2014). Podocalyxin is a marker of poor prognosis in colorectal cancer. BMC Cancer.

[22] Liu B, Liu Y, Jiang Y (2015). Podocalyxin promotes glioblastoma multiforme cell invasion and proliferation by inhibiting angiotensin-(1-7)/mas signalling. Oncology Rep.

[23] Povirk LF, Shuker DE (1994). DNA damage and mutagenesis induced by nitrogen mustards. Mutat Res.

[24] Colvin OM (1999). An overview of cyclophosphamide development and clinical applications. Curr Pharm Des.

[25] Rajski SR, Williams RM (1998). DNA cross-linking agents as antitumor drugs. Chem Rev.

[26] Kolesińska B, Drozdowska D, Kamiński ZJ (2008). The new analogues of nitrogen mustard with one, two or three 2-chloroethyloamino fragments. Reactions with nucleophiles. Acta Polon Pharm.

[27] Pomarnacka E, Bedarski P, Grunert R, Reszka P (2004). Synthesis and anticancer activity of novel 2-amino-4-(4-phenylpiperazino)-1,3,5-triazine derivatives. Acta Polon Pharm.

[28] Raval JP, Rai AR, Patel NH, Patel HV, Patel PS (2009). Synthesis and in vitro antimicrobial activity of N’-(4-(aryloamino)-6-(pirydyn-2-yloamino)-1,3,5,-triazyn-2-yl)benzo-hydrazide. Int J Chem Tech Res.

[29] Zacharie B, Abbott SD, Bienvenu JF, Cameron AD, Cloutier J, Duceppe JS, Ezzitouni A, Fortin D, Houde K, Lauzon C, Moreau N, Perron V, Wilb N, Asselin M, Doucet A, Fafard ME, Gaudreau D, Grouix B, Sarra-Bournet F, St-Amant N, Gagnon L, Penny CL (2010). 2,4,6-trisubstituted triazines as protein a mimetics for the treatment of autoimmune diseases. J Med Chem.

[30] Mandal S, Berube G, Asselin E, Mohammad I, Richardson VJ, Gupta A, Pramanik SK, Williams AL, Mandal SK (2007). A novel series of potent cytotoxic agents targeting G2/M phase of the cell cycle and demonstrating cell killing by apoptosis in human breast cancer cells. Bioorg Med Chem Lett.

[31] Kolesinska B, Barszcz K, Kaminski ZJ, Drozdowska D, Wietrzyk J, Switalska M (2012). Synthesis and cytotoxicity studies of bifunctional hybrids of nitrogen mustards with potential enzymes inhibitors based on melamine framework. J Enzym Inhib Med Chem.

[32] Frączyk J, Kolesińska B, Świątek M, Lipiński W, Drozdowska D, Kamiński ZJ (2016). Synthesis of arylo-1,3,5-triazines functionalized with alkylating 2-chloroethylamine fragments and studies of their cytotoxicity on the breast cancer MCF-7 cell line. Anti Cancer Agents Med Chem.

[33] Ventresca EM, Lecht S, Jakubowski P, Chiaverelli RA, Weaver M, Del Valle L, Ettinger K, Gincberg G, Priel A, Braiman A, Lazarovici P, Lelkes PI, Marcinkiewicz C (2015). Association of p75(NTR) and α9β integrin s modulates NGF-dependent cellular responses. Cell Signal.

[34] Carmichael J, DeGraff WG, Gazdar AF, Minna JD, Mitchell JB (1987). Evaluation of a tetrazolium-based semiautomated colorimetric assay: assessment of chemosensitivity testing. Cancer Res.

[35] Krętowski R, Kusaczuk M, Naumowicz M, Kotyńska J, Szynaka B, Cechowska-Pasko M (2017) The effects of silica nanoparticles on apoptosis and autophagy of glioblastoma cell lines. Nanomaterials (Basel) 21: 7(8) pii: E230. 10.3390/nano7080230 10.3390/nano7080230 PMC5575712 28825685

[36] Morgan LL (2015). The epidemiology of glioma in adults: a "state of the science" review. Neuro-Oncology.

[37] Dao P, Lietha D, Etheve-Quelquejeu M, Garbay C, Chen H (2017). Synthesis of novel 1,2,4-triazine scaffold as FAK inhibitors with antitumor activity. Bioorg Med Chem Lett.

[38] Dao P, Jarray R, Le Coq J, Lietha D, Loukaci A, Lepelletier Y, Hadj-Slimane R, Garbay C, Raynaud F, Chen H (2013). Synthesis of novel diarylamino-1,3,5-triazine derivatives as FAK inhibitors with anti-angiogenic activity. Bioorg Med Chem Lett.

[39] Sarkaria JN, Kitange GJ, James CD, Plummer R, Calvert H, Weller M, Wick W (2008). Mechanisms of chemoresistance in malignant glioma. Clin Cancer Res.

[40] Fu DJ, Song J, Hou YH, Zhao RH, Li JH, Mao RW, Yang JJ, Li P, Zi XL, Li ZH, Zhang QQ, Wang FY, Zhang SY, Zhang YB, Liu HM (2017). Discovery of 5,6-diaryl-1,2,4-triazines hybrids as potential apoptosis inducers. Eur J Med Chem.

[41] Krętowski R, Borzym-Kluczyk M, Stypułkowska A, Brańska-Januszewska J, Ostrowska H, Cechowska-Pasko M (2016). Low glucose dependent decrease of apoptosis and induction of autophagy in breast cancer MCF-7 cells. Mol Cell Biochem.

[42] Reed JC (2000). Mechanisms of apoptosis. Am J Pathol.

[43] Krętowski R, Borzym-Kluczyk M, Cechowska-Pasko M (2014). Efficient induction of apoptosis by proteasome inhibitor: bortezomib in the human breast cancer cell line MDA-MB-231. Mol Cell Biochem.

